# Activity-Based Sensing for Chemistry-Enabled Biology: Illuminating Principles, Probes, and Prospects for Boronate Reagents for Studying Hydrogen Peroxide

**DOI:** 10.1021/acsbiomedchemau.2c00052

**Published:** 2022-10-11

**Authors:** Marco S. Messina, Gianluca Quargnali, Christopher J. Chang

**Affiliations:** ^†^Department of Chemistry and ^‡^Department of Molecular and Cell Biology, University of California, Berkeley, Berkeley, California 94720, United States; § Department of Chemistry and Biochemistry, University of Delaware, Newark, Delaware 19716, United States

**Keywords:** Activity-based sensing, hydrogen peroxide, redox signaling, boronate, molecular imaging, in vivo imaging, fluorescence imaging, photoacoustic imaging, labeling, neurodegeneration

## Abstract

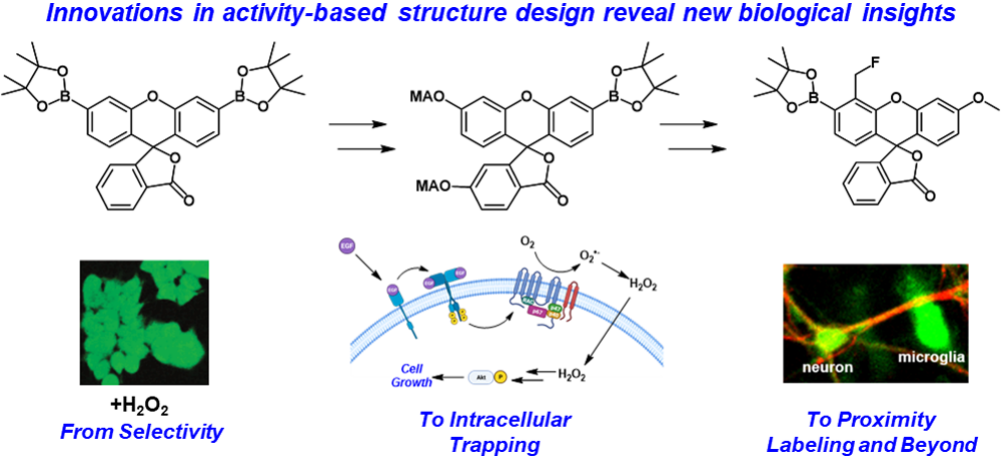

Activity-based sensing (ABS) offers a general approach that exploits chemical reactivity as a method for selective detection and manipulation of biological analytes. Here, we illustrate the value of this chemical platform to enable new biological discovery through a case study in the design and application of ABS reagents for studying hydrogen peroxide (H_2_O_2_), a major type of reactive oxygen species (ROS) that regulates a diverse array of vital cellular signaling processes to sustain life. Specifically, we summarize advances in the use of activity-based boronate probes for the detection of H_2_O_2_ featuring high molecular selectivity over other ROS, with an emphasis on tailoring designs in chemical structure to promote new biological principles of redox signaling.

Chemical sensors are powerful tools to illuminate new biology by enabling tracking of elemental species as well as small and large biomolecules in space and time.^1−3^ Indeed, cells orchestrate the many complex tasks required to maintain life by means of signaling pathways mediated by metal ions (Ca^2+^, Na^+^, K^+^, Cu^1+/2+^, Fe^2+/3+^, Zn^2+^), small-molecule and peptide/protein hormones and cytokines, lipids, glycans, and many other chemical signaling agents.^4−8^ In this context, reactive oxygen species (ROS) can also act as cellular messengers and play essential roles in redox signaling processes.^4,9−15^ ROS is an umbrella term used to denote a large array of small and transient molecular oxygen species, including the hydroxyl radical (•OH), ozone (O_3_), peroxynitrite (ONOO^–^), superoxide (O_2_^•–^), and hydrogen peroxide (H_2_O_2_). Historically, ROS have been viewed as dangerous byproducts of respiration, and indeed redox misregulation and resulting oxidative stress and damage events are implicated in many pathologies such as neurodegeneration, cancer, and autoimmune disorders.^16−18^ However, we now recognize that the chemistry and biology of ROS is much more sophisticated, as the generation of specific ROS is tightly regulated and also essential for normal signaling and metabolic functions.^10,12,13,19^

Along these lines, H_2_O_2_ is a particularly privileged redox signal produced from O_2_ by specific enzymatic sources, including NADPH oxidases, superoxide dismutases, and mitochondrial electron transport chain complexes.^13,20−22^ The relatively high stability of H_2_O_2_, compared to other ROS, allows it to traverse within and between cells and to regulate the activity of specific protein targets via cysteine or methionine oxidation, along with other emerging redox modifications.^11,23^ As such, H_2_O_2_ has been identified as a vital mediator of biological processes spanning immune response, angiogenesis, and cell proliferation, differentiation, and migration.^11−13,24−26^ This broad biology motivates the development of new chemical tools that enable selective tracking of biological H_2_O_2_ fluxes and gradients.^9^ Here, we provide an overview of the concept and application of activity-based sensing (ABS) as a general chemical approach for the study of biological messengers with molecular specificity. In particular, we focus on advances in the invention of boronate-based reagents by our laboratory and others that enable specific detection of hydrogen peroxide to illustrate the power of tailoring probe design to discover and decipher new biological principles. This Review provides a roadmap to inform future work and expand the ABS approach for a broader range of biological and environmental chemical analytes for study.

## Solving the Selectivity Challenge: Activity-Based Boronate Probes for Selective H_2_O_2_ Detection

**Figure 1:**
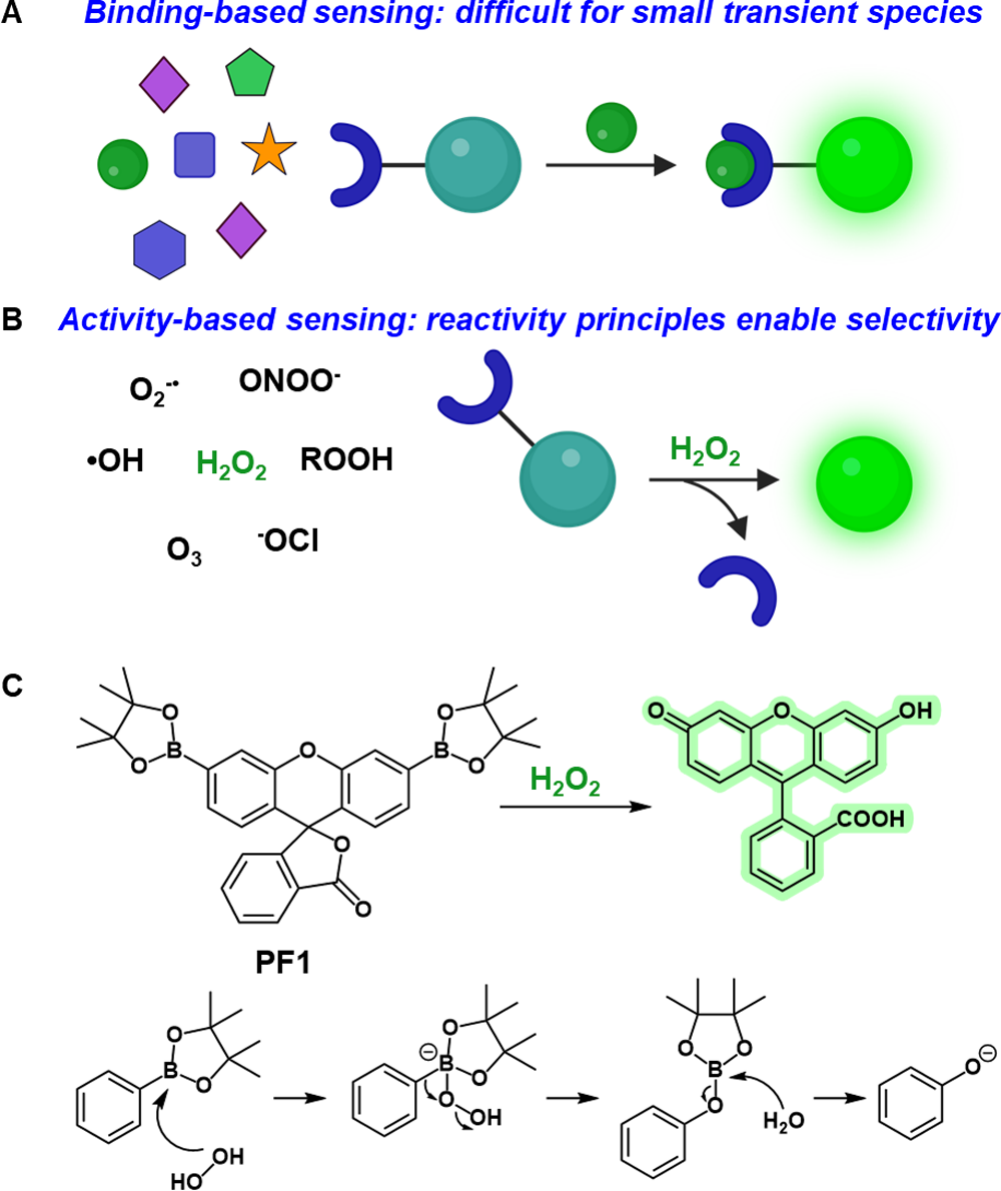
(A) Schematic cartoon depicting prototypical binding-based approaches for analyte sensing. (B) Schematic cartoon depicting activity-based sensing approaches which rely on chemical reactivity to enhance selectivity for transient species. (C) A primary example of activity-based sensing illustrated by the H_2_O_2_-mediated uncaging of aryl boronate groups on Peroxyfluor-1 (PF1) to generate fluorescein, with a mechanism for boronate-to-phenol conversion.

Selectivity is the primary challenge in initiating and implementing successful bioimaging design strategies. In this context, synthetic small-molecule probes offer an attractive complement to large-molecule sensors based on proteins and nucleic acids, as the former can be rapidly deployed across multiple cellular and organismal models without further specimen manipulation. Indeed, in the context of selective ROS detection, fluorescent protein sensors, including the HyPer and roGFP2 series, have contributed to our understanding of cellular H_2_O_2_ trafficking,^12,27−30^ but these reagents require genetic encoding and additional technical expertise for introduction. Early molecular probes, such as 2′,7′-dichlorodihydrofluorescein (DCFH), suffered from nonselective reactivity within the complex biological milieu.^1,9,13,31^ Additionally, the well-established and traditional binding-based approaches to fluorophore design, were not amenable for tracking small transient species such as ROS (Figure 1A).

**Figure 2:**
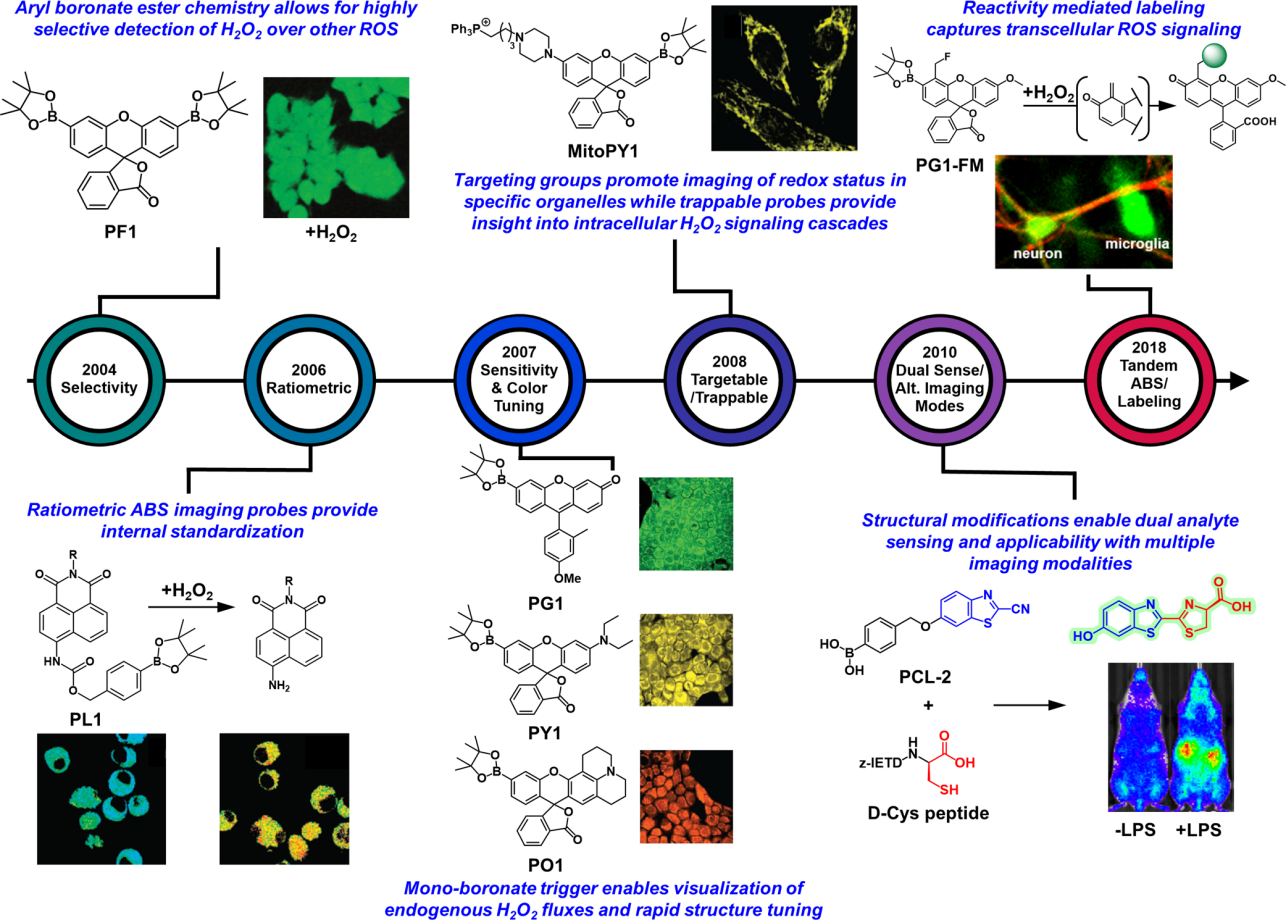
Chronological overview detailing how advances in the development of activity-based sensing (ABS) probes in H_2_O_2_ detection lead to new biological discovery of redox signaling principles, emphasizing the synergy between ABS chemical probe design and biological experiments. Adapted with refs (32, 46, 55, 59, and 96). Copyright 2004, 2008, 2010, 2008, and 2013 American Chemical Society, respectively. Adapted with permission from ref (133). Copyright 2021 National Academy of Sciences.

As such, our laboratory initiated a research program focused on harnessing chemical reactivity as a platform to track small molecule transient analytes in biologically relevant settings, opening a field we term “activity-based sensing” (ABS) (Figure 1B).^1,32^ With the introduction of Peroxyfluor-1 (PF1), we established boronate uncaging as a viable design strategy for selective H_2_O_2_ sensing in biologically relevant settings (Figures 1C and 2). Inspired by organic chemistry reported in the 1950s showing that boronates could be converted to phenols by H_2_O_2_-mediated oxidation, PF1 was based on the traditional fluorescein scaffold but modified with aryl boronate esters at the xanthenone 3′ and 6’ positions.^32−34^ In this form, the probe adopted the closed and nonfluorescent lactone form (Figure 1C). Oxidative deprotection of the aryl boronate esters to the corresponding phenolates generates the open quinoid and fluorescent form of fluorescein, thus enabling the selective detection of H_2_O_2_ in live-cell models.^32^ Two key features of the boronate oxidation reaction mechanism are (1) its two-electron nature, which distinguishes peroxide over radical ROS that act as single-electron oxidants, and (2) the elimination of water, which enables kinetic discrimination over alkyl peroxides, where alcohol would be the leaving group. When used in conjunction with appropriate controls such as nitric oxide synthase inhibitor to eliminate possibilities of peroxynitrite detection, which is generated at much lower levels with a much shorter lifetime if it is present, boronate probes are reliable activity-based reagents for selective H_2_O_2_ bioimaging.^1,35,36^

The success of PF1 and ease of aryl boronate ester installation onto virtually any type of fluorophore scaffold has facilitated the rapid adoption of this ABS strategy by the broader chemical biology community. Indeed, the field has witnessed an influx of highly creative fluorophore structures for H_2_O_2_ sensing that span the visible and near-infrared (near-IR) wavelength spectrum, afford ratiometric readouts, target specific organelles, and simultaneously monitor other important analytes (Figure 2). Importantly, each design advance in new chemical structures for ABS has unearthed new fundamental insights into biological H_2_O_2_ signaling, from the discovery of transmembrane proteins that facilitate H_2_O_2_ uptake intracellularly to identifying specific subcellular sources of ROS in conjunction with proteomic analysis.^37,38^ We direct the reader to other excellent reviews should they seek detailed historical contexts for ABS, extensive catalogues on photophysical properties of ABS probes, and/or discussions on ABS of other analytes.^1,39−41^

## Ratiometric Activity-Based Sensing Probes Enable Self-Calibration by Two-Color Responses

Soon after the development of PF1, we sought to rapidly expand the scope and generality of activity-based probe design to enable ratiometric fluorescence imaging of H_2_O_2_. Indeed, fluorescence quantification of a single emission component is challenging. In particular, potential variations in instrument hardware and parameters, photobleaching, probe aggregation, and variations (pH, polarity, temperature, etc.) in the surrounding probe microenvironment, among other factors, may lead to confounding and even irreproducible results.^42,43^ Ratiometric fluorescence imaging can bypass many of these challenges due to the internal referencing provided by simultaneously measuring changes in the fluorescence intensity between two emission bands and calculating their ratio.

**Figure 3:**
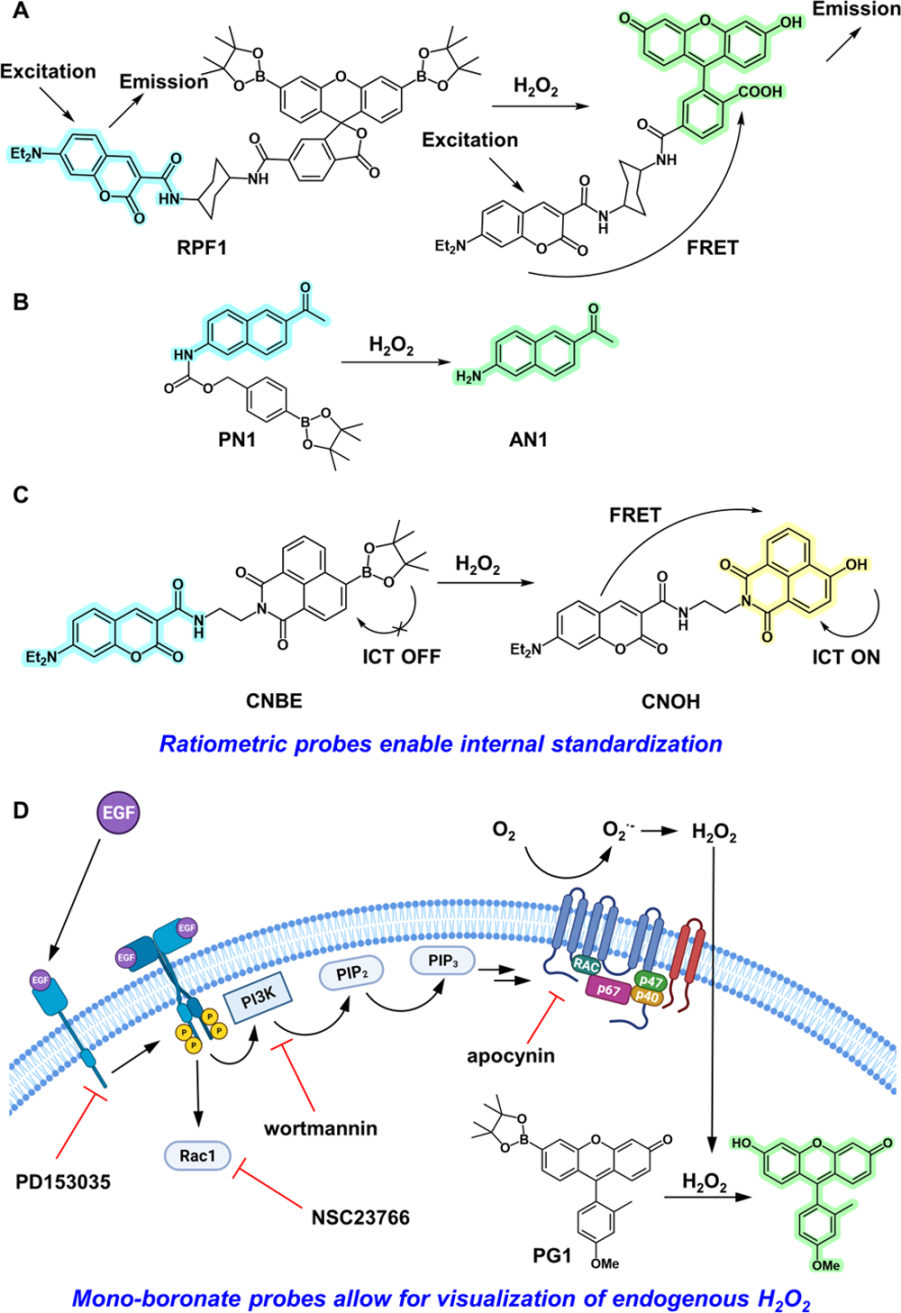
(A) Schematic detailing H_2_O_2_-mediated uncaging of Ratio-Peroxyfluor-1 (RPF1), which relies on a fluorescence resonance energy transfer (FRET)-based mechanism for ratiometric signal generation. (B) Schematic depicting H_2_O_2_-mediated uncaging of PN1. (C) CNBE generates CNOH in the presence of H_2_O_2_ which takes advantage of both internal charge transfer (ICT) and FRET mechanisms to produce a blue-to-yellow ratiometric signal. (D) Peroxy Green-1 (PG1) exhibits greatly enhanced sensitivity, thus enabling H_2_O_2_ imaging and deciphering of components of the peroxide signaling pathways during growth factor stimulation. The NADPH oxidase (Nox) inhibitor apocynin, PI3K inhibitor wortmannin, Rac1 inhibitor NSC23766, and epidermal growth factor (EGF) inhibitor PD153035 all diminish H_2_O_2_ signaling.

In initial studies to achieve this goal, we developed Ratio-Peroxyfluor-1 (RPF1) as a Förster resonance energy transfer (FRET)-based approach toward ratiometric ABS imaging (Figure 3A).^44^ We drew inspiration from previous work by Nagano and co-workers, in which they employed FRET-based fluorophore systems to measure protein tyrosine phosphatase (PTP) activity by tuning spectral overlap integrals.^45^ RPF1 is composed of both coumarin and aryl boronate ester protected fluorescein scaffolds linked together by a short cyclohexane spacer (Figure 3A).^44^ In its protected form, fluorescein remains locked in the lactone form and does not exhibit an overlapping absorption band with the coumarin emission, effectively shutting off FRET-based fluorescence. H_2_O_2_-mediated aryl boronate ester deprotection generates the open quinoid form of fluorescein thereby promoting spectral overlap between coumarin emission and fluorescein absorption thus enabling FRET-based fluorescence (Figure 3A). RPF1 was highly selective for H_2_O_2_ over other ROS and reactive nitrogen species (RNS) and enabled quantification of H_2_O_2_ production in isolated yeast mitochondria upon antimycin A stimulation.

In order to translate ratiometric activity-based imaging to live-cell models, we developed Peroxy Lucifer 1 (PL1, Figure 2), which leverages an intramolecular charge transfer (ICT) mechanism to generate a ratiometric fluorescence response.^46^ The naphthalimide core is rendered electron-poor when the carbamate-tethered aryl boronate cage is intact with a maximum fluorescence emission centered at 475 nm. H_2_O_2_-mediated deprotection exposes the free amine, thus promoting greater electron donation into the core naphthalimide scaffold and resulting in a new fluorescence maximum at 540 nm. This blue-to-green fluorescent response shift enabled the ratiometric analysis of endogenous H_2_O_2_ bursts produced and localization of these pools in PMA-stimulated RAW 264.7 macrophages.^46^ In collaboration with Cho’s laboratory, we also established Peroxy Naphthalene 1 (PN1) for deep tissue ratiometric imaging via two-photon microscopy, which provided the capability to monitor differences in basal H_2_O_2_ levels in distinct regions of rat hippocampal slices (Figure 3B).^47^

A particularly elegant ratiometric activity-based H_2_O_2_ sensor developed by Lin and co-workers couples both ICT and FRET mechanisms. The sensor dyad is composed of a coumarin scaffold linked to a boronate-modified napthalimide (CNBE) (Figure 3C).^48^ Deprotection of CNBE by H_2_O_2_ to generate CNOH activates ICT, resulting in a bathochromically shifted naphthalimide absorbance that overlaps with coumarin emission (Figure 3C). This strategy was capable of tracking H_2_O_2_ elevations in lipopolysaccharide (LPS)-stimulated HeLa cells and *in vivo* zebrafish models.^48^ Other recent examples of ratiometric probes for activity-based peroxide sensing have been applied to monitor oxidative stress during ischemic brain injury or in Alzheimer’s disease (AD) and Parkinson’s models.^39,49,50^ As many of these systems utilize organelle targeting fragments or sense multiple analytes in addition to providing a ratiometric response, we elaborate on them in subsequent sections (*vide infra*).

## Monoboronate Activity-Based H_2_O_2_ Probes for Increased Sensitivity and Modular Color Palette Tuning

Although PF1 and related bis-boronate protected probes developed subsequently provided an important foundation for the ABS of H_2_O_2_ with high ROS specificity, these reagents lacked the sensitivity required to monitor endogenous variations in H_2_O_2_ levels.^32,44,51^ This situation arises because 2 equiv of H_2_O_2_ is necessary to generate one uncaged fluorophore. To overcome this limitation, our laboratory introduced the monoboronate Peroxy Green 1 (PG1) and Peroxy Crimson 1 (PC1) probes based on Tokyo Green and resorufin scaffolds, respectively (Figure 3D).^35,52^ Both PG1 and PC1 exhibited greater fluorescence emission intensities upon uncaging than the previously reported diboronate analogues and retained high selectivity toward H_2_O_2_ over other ROS and RNS. Notably, these new monoboronate protected analogues also outperformed the nonselective 2′,7′-dichlorodihydrofluorescein (DCFH) probe that was traditionally employed in cellular peroxide assays. These gains in sensitivity enabled the use of PG1 to monitor endogenous H_2_O_2_ elevations in growth factor signaling, as illustrated in live A431 cells stimulated by epidermal growth factor (EGF, Figure 3D). The high levels of epidermal growth factor receptors (EGFR) expressed on A431 cells provide a useful biological model for H_2_O_2_ signaling. Indeed, stimulation via EGF promotes H_2_O_2_ production via the Nox/phosphoinositide 3-kindase (PI3K) pathway (Figure 3D).^35,53^ Deployment of small molecule inhibitors of either the EGFR kinase domain (PD153035), phosphatidylinositol-3-OH kinase (wortmannin), or Nox (apocynin) attenuated the fluorescence intensity observed in PG1 stained A431 cells due to decreased H_2_O_2_ production (Figure 3D). Additionally, PG1 stained A431 cells treated with the NO synthase inhibitor L-N^G^-nitroarginine methyl ester (L-NAME) maintained similar fluorescence responses to EGF-stimulated cells, showing that the boronate probe is indeed detecting H_2_O_2_ and not peroxynitrite in this biological context. Taken together, these experiments provided the first direct imaging evidence for H_2_O_2_-selective signaling in live cells. Importantly, PG1 staining enabled visualization of H_2_O_2_ in postnatal rat hippocampal neurons suggesting that similar pathways of ROS signaling are also active in brain systems.^35^

In addition to enhanced sensitivity, the monoboronate platform permitted a greater degree of flexibility in fluorophore design, as one-half of the molecule was now free to modify to introduce other functionalities. This characteristic enabled rapid incorporation of the monoboronate trigger into rhodol-based scaffolds constructed from aminophenol (Peroxy Emerald 1, PE1), diethylaminophenol (Peroxy Yellow 1, PY1), and julolidine (Peroxy Orange 1, PO1) components (Figure 2). Facile access to this large color palette enabled dual-color imaging and simultaneous detection of two distinct ROS in RAW264.7 macrophages when coupled with the previously reported 2-[6-(4′-amino)phenoxy-3*H*-xanthen-3-on-9-yl]benzoic acid (APF) probe which responds predominately to HOCl, but also peroxynitrite and the hydroxyl radical.^54,55^ Notably, this study showed that the boronate reagents do not give a turn-on response to HOCl in cellular contexts.

## Targetable and Trappable Activity-Based Sensing Probe Derivatives for Single-Cell and Subcellular H_2_O_2_ Imaging

Building on the high degree of tunability regarding photophysical properties and molecular selectivity toward H_2_O_2_, a next set of advances have led to improvements in signal retention and/or spatial resolution through the introduction of organelle-targeting groups and/or cell-trapping mechanisms. Indeed, the ability to study perturbations in H_2_O_2_ production and trafficking in pathological contexts with organellar resolution remains an ongoing and critical challenge to meet in the redox biology field.

**Figure 4:**
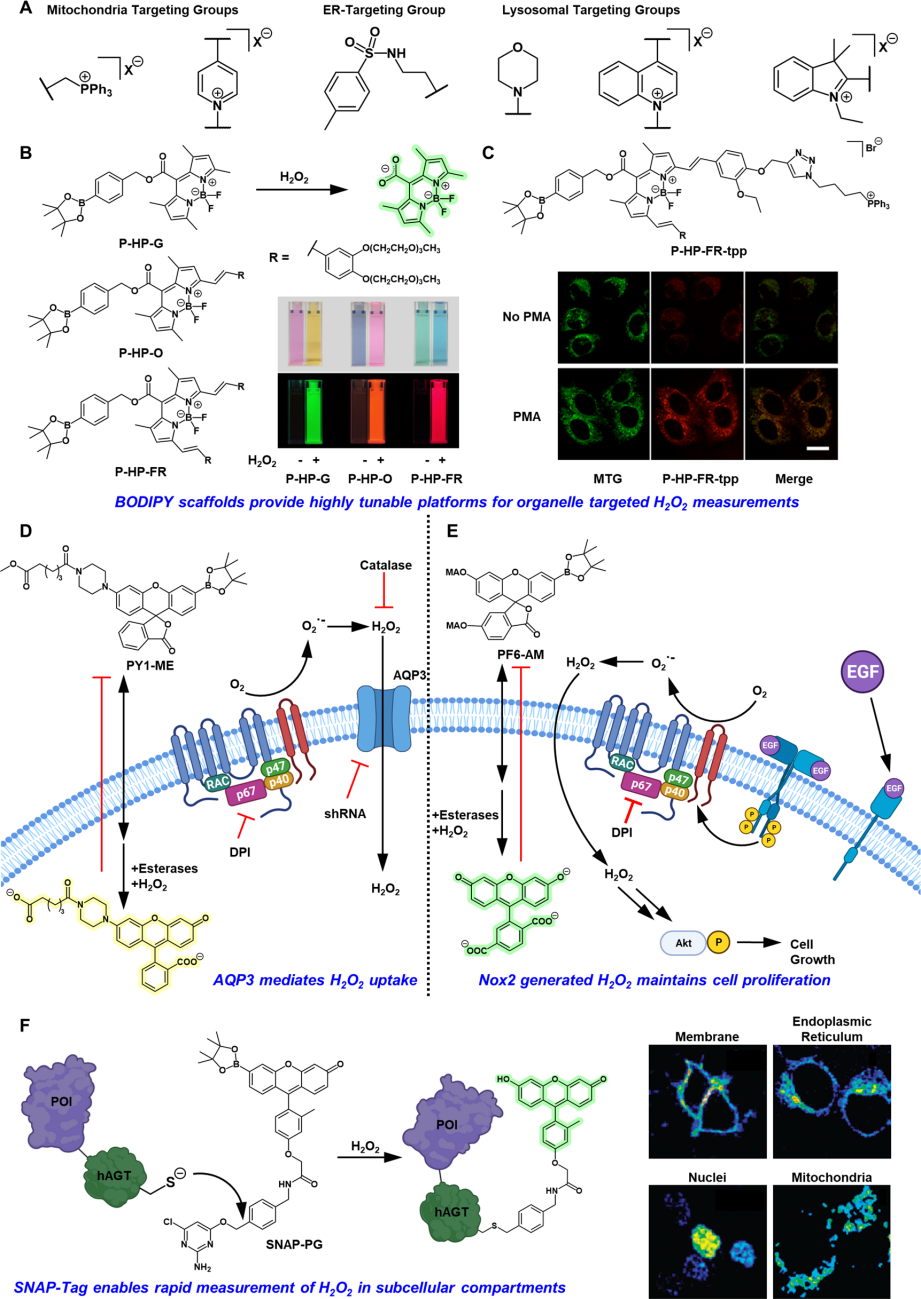
(A) Commonly used fragment warheads for organelle targeting of activity-based sensing probes. (B) *meso*-Protected BODIPY dyes which undergo H_2_O_2_-mediated uncaging to elicit fluorescence responses across the visible wavelength window. (C) Structure of mitochondria targeting P-HP-FR-tpp and live cell fluorescence confocal imaging in PMA stimulated HeLa cells. Adapted from ref (60). Copyright 2017 American Chemical Society. (D) Schematic representing how the development of the cell-trappable probe Peroxy Yellow-1 Methyl Ester (PY1-ME) enabled our laboratory to identify aquaporins (AQPs), such as AQP3, also serve as membrane channels regulating intracellular H_2_O_2_ uptake. (E) Schematic demonstrating studies with the cell-trappable probe Peroxyfluor-6 Acetoxymethyl Ester (PF6-AM), which identified the essential role of H_2_O_2_ production via Nox2 in neural stem cell proliferation. (F) Protein-targeting strategies, such as utilization of SNAP-tag technology, can direct H_2_O_2_ sensors to specific organelles by genetic encoding. Adapted with permission from ref (81). Copyright 2010 American Chemical Society.

The accumulation of molecular cargo to specific cellular organelles can be promoted by the attachment of robust targeting scaffolds. For example, owing to their combination of positive charge and lipophilicity, triphenylphosphonium (TPP) groups are often used for mitochondrial targeting, while morpholino substitution as a basic amine enhances lysosomal accumulation, and methyl sulfonamide groups can target the endoplasmic reticulum (ER) (Figure 4A).^56−58^ In this context, our laboratory developed MitoPY1, which harbors both a pendant TPP group and the aryl boronate ester trigger on a rhodol scaffold as a first-generation, organelle-targeting activity-based sensing probe (Figure 2).^59^ MitoPY1 imaging established its ability to monitor mitochondrial H_2_O_2_ pools across multiple cell types, including HeLa, HEK293T, Cos7, and CHO.K1. Moreover, the probe enabled H_2_O_2_ visualization in paraquat-stimulated HeLa cells, a commonly used *in vitro* Parkinson’s disease model that induces mitochondrial oxidative stress.^59^ Boron dipyrromethene (BODIPY) dyes have also found extensive use in mitochondrial H_2_O_2_ detection, as the BODIPY scaffold is highly tunable and relatively inert to pH and polarity fluctuations.^60^ Li and co-workers tethered H_2_O_2_-responsive triggers onto the *meso*-position of BODIPY structures which rendered the probes nonfluorescent due to the electron-withdrawing nature of the triggers (Figure 4B, C). Uncaging in aqueous solutions generates the free *meso*-carboxylate anion and a substantial increase in fluorescence quantum yield.^61^ Multiple derivatives (P-HP-G, P-HP-O, P-HP-FR) spanning the visible wavelength spectrum were synthesized, including a TPP-modified mitochondria targeting derivative (P-HP-FR-tpp), which was able to visualize endogenous H_2_O_2_ fluxes in live-cell models (Figure 4B, C). Additionally, the highly tunable BODIPY scaffold also enabled the creation of H_2_S and protease responsive analogues.^60^ The TPP mitochondrial targeting strategy has also been used to develop two-photon fluorescent probes for ratiometric imaging of H_2_O_2_ in tissue specimens.^62^ The Cho and Kim groups took advantage of a carbamate-linked aryl boronate trigger to synthesize SHP-Mito, which exhibited a fluorescence maximum at 470 nm. Akin to what is observed for PL1, more favorable ICT resulting from H_2_O_2_-promoted uncaging shifts the fluorescence maximum to 540 nm, giving rise to a ratiometric response.^46^ Two-photon microscopy of rat hippocampal slices incubated with SHP-Mito and treated with H_2_O_2_ show the ability to visualize H_2_O_2_ at 180 μm depths.^62^ The development of ABS fluorophores with greater two-photon absorption cross sections and increased sensitivity toward variations in endogenous H_2_O_2_ should enable further advances in activity-based imaging via two-photon microscopy.

Beyond TPP, mitochondrial-targeting of H_2_O_2_ ABS fluorophore systems based on quinolinium, indolinium, or pyridinium quaternary ammonium salt frameworks have been reported.^63−69^ Tang and co-workers have made use of both the indolinium quaternary ammonium salt and methyl sulfonamide groups to develop both mitochondria- and ER-directed probes, respectively.^58^ As each fluorophore exhibited distinct fluorescence emission maxima, the authors were able to concurrently track variations in H_2_O_2_ levels within each organelle. Applying these probes to live-cell models of apoptosis, the authors found that treatment with apoptotic stimulants such as l-buthionine sulfoximine (BSO), carbonyl cyanide *m*-chlorophenylhydrazone (CCCP), and Tm all increased organellar H_2_O_2_ levels at different rates, due to the different mechanisms of action for each stimulant. Notably, treatment of cells with CCCP, which directly targets the mitochondria, resulted in sustained H_2_O_2_ elevations in both organelles after an initial mitochondrial H_2_O_2_ burst, indicating the interplay between the two organelles. Whereas treatment of cells with Tm, an ER-targeting stimulant, demonstrated a sharp rise in ER H_2_O_2_ levels with a delayed sharp rise in mitochondrial H_2_O_2_ hinting toward a diffusive process.^58^ Indeed, multianalyte measurement with subcellular-targeted probes can afford exciting new opportunities to further investigate mechanisms of interorganellar ROS communication. However, the simultaneous deployment of two single-analyte responsive probes should be approached with caution, as differences in permeability and/or photophysical properties of individual probes can potentially confound results in the absence of proper control experiments.

Morpholine substitution is frequently utilized to enhance lysosomal accumulation of fluorescent dyes.^70−72^ Indeed, systems developed for H_2_O_2_ sensing have found use in two-photon microscopy for deep tissue imaging of *ex vivo* rat liver tissue and in live-cell models of ischemia/reperfusion.^70,71^ A pH-switchable spirobenzopyran-based fluorophore that elicits a turn on response only at low pH and in the presence of H_2_O_2_ (HP-L1) has also been reported.^73^ This probe does not contain a lysosomal-targeting group, but its response is only visible in lysosomes due to decreased pH within the organelle.

We have also identified Nuclear Peroxy Emerald 1 (NucPE1) to monitor H_2_O_2_ fluxes in cell nuclei, though the mechanism that promotes nuclear localization remains unclear.^74^ NucPE1 was applied to *in vivo* zebrafish models of aging by overexpressing Sir-2.1, which is an NAD-dependent histone deacetylase known to increase zebrafish lifespan. Using NucPE1, we demonstrated that Sir-2.1-overexpressing zebrafish exhibited lower basal levels of H_2_O_2_ and were less sensitive toward exogenous H_2_O_2_ treatment over their wild-type counterparts.^74^ The attachment of peptides to ABS probes also allows for facile tuning of targeting capabilities. Examples of this strategy include the Nuclear Localization Signal peptide for nuclear targeting, the KRGD peptide sequence to selectively target ovarian cancer cells, and octreotide for somatostatin receptor binding.^75−77^ Ester protection of carboxyl or phenolic hydroxyl groups on molecular cargo is known to increase lipophilicity, thereby enhancing cell permeability.^78^ Once inside the cell, deprotection via native intracellular esterases exposes the free carboxylic acid or phenol groups, effectively trapping the compound through polarity reversal. These features can be enhanced by increasing the number of ester-protected sites.^79^ To leverage this strategy, we synthesized both Peroxy Yellow 1 Methyl-Ester (PY1-ME) and Peroxyfluor-6 acetoxymethyl ester (PF6-AM) concurrently (Figure 4D, E).^37,80^ Deployment of PY1-ME in flow cytometry and confocal fluorescence imaging experiments enabled us to identify that specific aquaporin (AQP) isoforms, AQP3 and AQP8, are involved in shuttling H_2_O_2_ from the extracellular space to the intracellular cytosol (Figure 4D). This work demonstrates that H_2_O_2_ transport through membranes is not passive, as was previously believed. This belief was due to the ability of NO, another transient small-molecule signal, to pass freely through membranes. Instead, H_2_O_2_ transport is regulated via specific channels. Indeed, aquaporins are pervasive transmembrane channel proteins that facilitate the uptake of water and other small molecules intracellularly, and H_2_O_2_ can be viewed as a larger water-like substrate. Western blot analysis of Akt phosphorylation, coupled with complementary HyPer-based H_2_O_2_ imaging in EGF-stimulated cell models, established a biochemical pathway that links EGF activation of Nox to generate H_2_O_2_, with subsequent intracellular uptake via AQP3 to the promotion of downstream Akt signaling cascades.

Tandem efforts from our laboratory were devoted toward applying PF6-AM imaging to elucidate the role of H_2_O_2_ in redox-driven signaling pathways in the brain, with a focus on neural stem cells and neurogenesis (Figure 4E). PF6-AM imaging, coupled with measurements of Akt and Erk phosphorylation, revealed that a related growth factor stimulation pathway involving Nox2-generated H_2_O_2_ promotes growth and proliferation of adult hippocampal stem/progenitor cells (AHPs), leading to more neurogenesis. In this biochemical signaling cascade, Nox-derived H_2_O_2_ serves as a writer on the phosphatase PTEN, a key regulatory target that inhibits kinase Akt signaling. Cysteine oxidation of PTEN by the H_2_O_2_ signal results in temporary inactivation of its activity and reciprocal amplification of Akt signaling and growth/proliferation pathways. Negative control experiments via chemical inhibition or shRNA knockout models of key components of this pathway strengthen these findings (Figure 4E). Additionally, *in vivo* mouse models using Nox2 knockout (Nox2^–/–^) mice and CL57BL/6J control mice established the pivotal role of Nox2-generated H_2_O_2_ in promoting neurogenesis, showing that peroxide production by this pathway represents a quarter to a third of the neurogenesis observed in the murine brain.^80^ Thus, coupling the esterase-mediated cell trapping mechanism with highly sensitive ABS boronate probes enabled us to reveal key components in fundamental intracellular signaling mechanisms of H_2_O_2_.

Our laboratory has also utilized SNAP-tag fusion protein technology to direct H_2_O_2_-responsive probes to specific subcellular compartments (Figure 4F).^81^ The SNAP-tag fusion technology pioneered by Johnsson and co-workers enables the transfer of molecular cargo to proteins of interest via the development of fusion proteins with *O*^6^-alkylguanine-DNA alkyltransferase (hAGT), which undergoes covalent labeling with *O*^6^-benzylguanine derivatized cargo (Figure 4F).^82,83^ We appended a Peroxy Green analogue onto both a membrane-permeable SNAP substrate for intracellular labeling and membrane-impermeable derivative for cell surface labeling (SNAP-PG, Figure 4F). Application of SNAP-PG probes to live mammalian cells expressing the SNAP tag at the cell membrane, or in the nuclei, mitochondria, or endoplasmic reticulum facilitated the site-specific imaging of H_2_O_2_ at each location with high precision as measured in colocalization staining experiments.^81^ Thus, this platform combines the significant targeting capabilities of genetic encoding with the high selectivity afforded by ABS approaches and offers a compelling approach to monitoring subcellular H_2_O_2_ distribution in pathological models.

## Dual Sensing and Alternative Imaging Modalities to Map Peroxide across Multiple Biological Length Scales

The development of single probes able to respond to multiple analytes was a significant advance in the activity-based sensing field.^84,85^ Indeed, multianalyte sensing can generate new insights on the complex interplay between different biological phenomena, as cellular metabolic and signaling pathways rely on the synergy of multiple chemical species. In the context of H_2_O_2_ sensing, many probes have been designed that simultaneously respond to hypochlorite,^86−88^ nitrous oxide,^89^ hydrogen sulfide,^90,91^ thiols,^92^ pH,^93−95^ enzymes,^96−98^ protein aggregates,^99,100^ and viscosity.^101−103^ Indeed, structure design for multianalyte sensing has been applied to prepare dual-locked prodrugs; these systems are beyond the scope of this review and covered extensively elsewhere.^39,104^ Dual-responsive probes hold some general advantages over the simultaneous use of single-analyte probes. Notably, differences in membrane permeability, localization, and metabolism of two probes can result in confounding results or data misinterpretation. Much like ratiometric reagents, dual-responsive probes often take advantage of modulating photoinduced electron transfer (PET), internal charge transfer (ICT), or Forster resonance energy transfer (FRET) processes, depending on the type of system that is being investigated.^105^

Our first foray into dual-analyte sensing was the creation of a dendrimer-based system composed of both SNARF2 and PF1.^93^ The fluorescence and emission exhibited by SNARF2 is modulated by pH, whereas PF1 provides an irreversible fluorescence turn-on response in the presence of H_2_O_2_, thus enabling simultaneous multiplex imaging of both pH and redox status. Application of this dendrimer system to immune stimulated RAW264.7 macrophage models of phagocytosis elucidated the role of Nox in pH regulation as pharmacological Nox inhibition during oxidative bursts resulted in phagosomal acidification.^93^ More recent examples of pH and H_2_O_2_ dual-responsive systems rely on single small-molecule probes and have been translated to in vivo LPS stimulated or H_2_O_2_ injected mouse models.^94,95^

**Figure 5:**
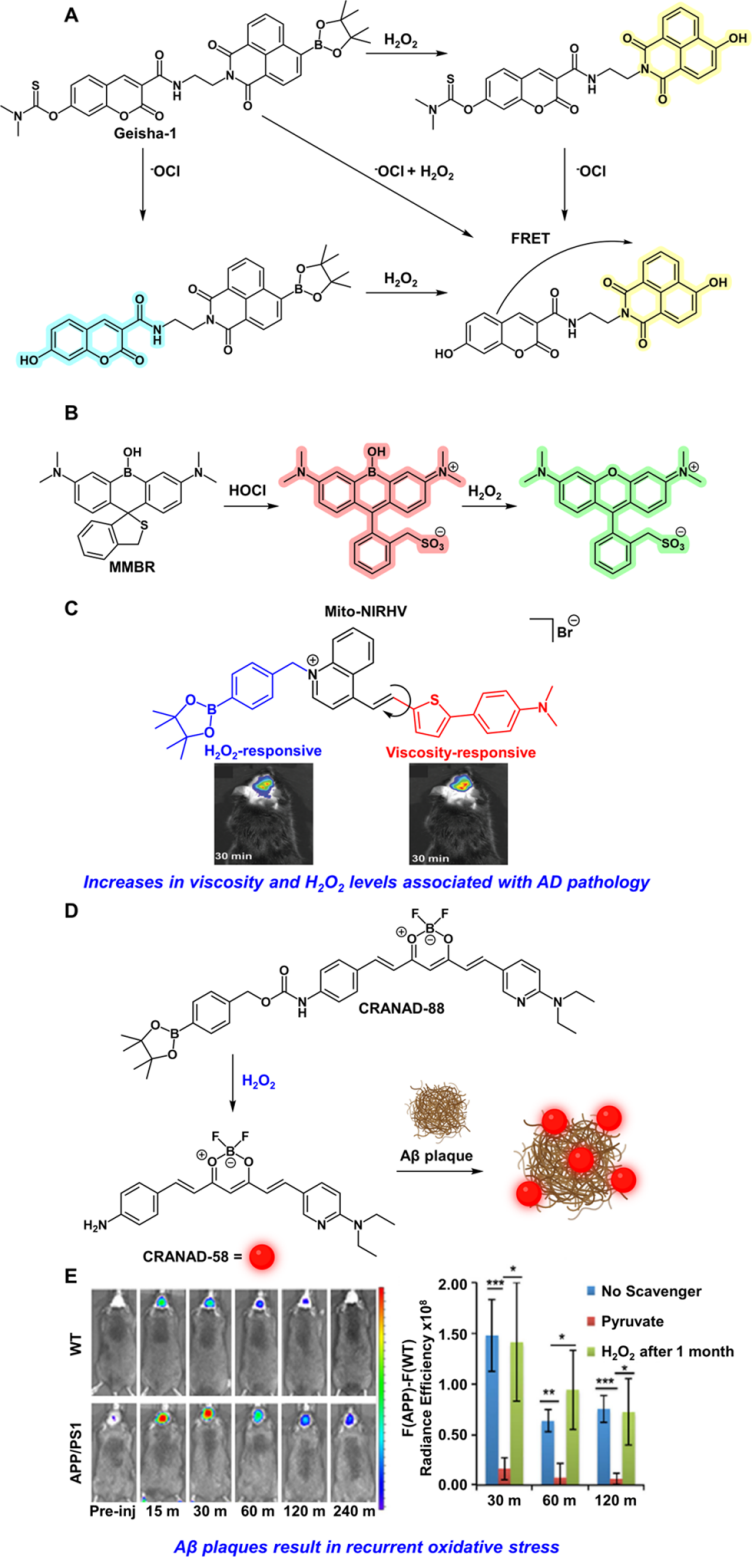
(A) Scheme depicting HOCl and H_2_O_2_ dual-sensing of Geisha-1. (B) Scheme depicting sequential HOCl and H_2_O_2_ mediated sensing by MMBR. (C) Mito-NIRHV simultaneously measures H_2_O_2_ and changes in viscosity and has been applied to in vivo mouse models of AD pathology. Adapted with permission from ref (101). Copyright 2020 The Royal Society of Chemistry. (D) CRANAD-88 responds to H_2_O_2_ to generate fluorescent CRANAD-58 which upon binding to Aβ plaques undergoes fluorescence amplification. (E) CRANAD-88 was applied to monitor both H_2_O_2_ and plaque formation in in vivo AD mouse models. Adapted with permission under a Creative Commons CC BY license from ref (100). Copyright 2016 Springer Nature.

HOCl is also an important target for sensor systems due to its involvement with H_2_O_2_ in immune response.^106^ As such, dual-responsive single molecule fluorophore systems have been designed to monitor both analytes simultaneously.^87^ Chen and co-workers devised a dual ICT and FRET-based strategy via the design of a thiocarbamate modified coumarin unit linked to an aryl boronate-modified naphthalimide (Geisha-1, Figure 5A).^86^ H_2_O_2_-mediated deprotection of the aryl boronate ester or thiocarbamate deprotection upon HOCl sensing results only in ICT with emission at distinct wavelengths (550 nm for H_2_O_2_ and 452 nm for HOCl). However, deprotection of both results in a FRET-based response with fluorescence emission at 550 nm.^86^ The Cui group also devised an elegant ratiometric strategy for simultaneous monitoring of HOCl and H_2_O_2_.^88^ Instead of an aryl boronate approach for H_2_O_2_ sensing, their design involves the use of a ratiometric B-rhodamine core scaffold with a mercaptomethyl HOCl sensor (MMBR, Figure 5B).^88^ Sensing of HOCl results in the probe assuming an open zwitterionic form and fluorescence emission centered at 646 nm. Subsequent reaction with H_2_O_2_ results in the formation of a rhodamine structure with fluorescence emission hypsochromically shifted to 575 nm. Reaction with only H_2_O_2_ results in a closed and nonfluorescent rhodamine structure, thus MMBR can only be used to visualize HOCl or both H_2_O_2_ and HOCl. Notably, beyond visualizing endogenous fluxes of both HOCl and H_2_O_2_ in live cell models of oxidative stress, MMBR was also able to monitor wound induced myeloperoxidase catalyzed conversion of H_2_O_2_ into HOCl in zebrafish models.^88^

Particularly interesting are dual-responsive probes applied to neurodegenerative disease models. Elegant reports from the Lin and Liu groups established Mito-VH and Mito-NIRHV probes, respectively, to monitor mitochondrial changes in both H_2_O_2_ and viscosity, as increases in both have been linked to Alzheimer’s disease (AD) pathology (Figure 5C).^101,102,107^ The intricate design of these probes utilize the mitochondrial targeting fragment as a strong electron acceptor, which when linked via an alkene tether to an electron donating group generates a donor-π-acceptor (D-π-A) system. Such a characteristic is commonplace in molecules using twisted intramolecular charge transfer (TICT) to modulate emission. As such, TICT in these systems is regulated via rotation of the electron donating fragment which varies with solution viscosity (Figure 5C). Both H_2_O_2_-mediated uncaging and increases in viscosity can be monitored through fluorescence turn-on responses at distinct wavelength regions, with H_2_O_2_ uncaging exhibiting blue-shifted emission in both instances.^101,102^ The addition of the thiophene spacer for Mito-NIRHV enabled NIR fluorescence emission for both sensors. As such, the Liu group applied Mito-NIRHV for the bioimaging of endogenous H_2_O_2_ and viscosity elevations in in vivo mouse models. Mice treated with LPS and genetic AD-model (APP/PS1 transgenic) mice demonstrated much stronger fluorescence responses over untreated and WT mice indicating the association between increased viscosity and H_2_O_2_ to AD pathology (Figure 5C).^101^ Fluorophores that monitor protein aggregates such as amyloid β (Aβ) have also been developed as Aβ and oxidative stress are both hallmarks of AD pathology. Ran and co-workers synthesized CRANAD-88, which utilizes a self-immolative ABS trigger that liberates CRANAD-58, a compound with high affinity for Aβ, in the presence of H_2_O_2_ (Figure 5D). As such, both chemical reactivity and substrate affinity contribute to the functioning of this probe, leading to amplified near-IR fluorescent readouts upon dual H_2_O_2_ sensing and Aβ binding. Employing CRANAD-88 in mouse models of AD, the authors found increased levels of oxidative stress in AD mice brains compared to WT mice (Figure 5E). Additionally, toxic H_2_O_2_ levels could be regenerated in the presence of Aβ plaques even after scavenging with sodium pyruvate, thus indicating that treatment options should focus on the simultaneous removal of Aβ and ROS (Figure 5E).^100^

In addition, there are many structural modifications in ABS fluorophore design that afford alternatives to fluorescence imaging and/or enable combining multiple imaging modalities. Systems making use of PET,^108^ photoacoustic,^109,110^ or chemi/bioluminescent^96,111−113^ readouts tend to benefit *in vivo* bioimaging through greater tissue depth penetration though sometimes at the expense of resolution. Early reports exploring alternative imaging modalities from our group made use of H_2_O_2_-responsive luminescent lanthanide compounds for time-gated imaging as a strategy for decreasing autofluorescence in biological specimens.^114^ We also developed PET-based approaches to H_2_O_2_ imaging in collaboration with the Wilson laboratory.^108^

**Figure 6:**
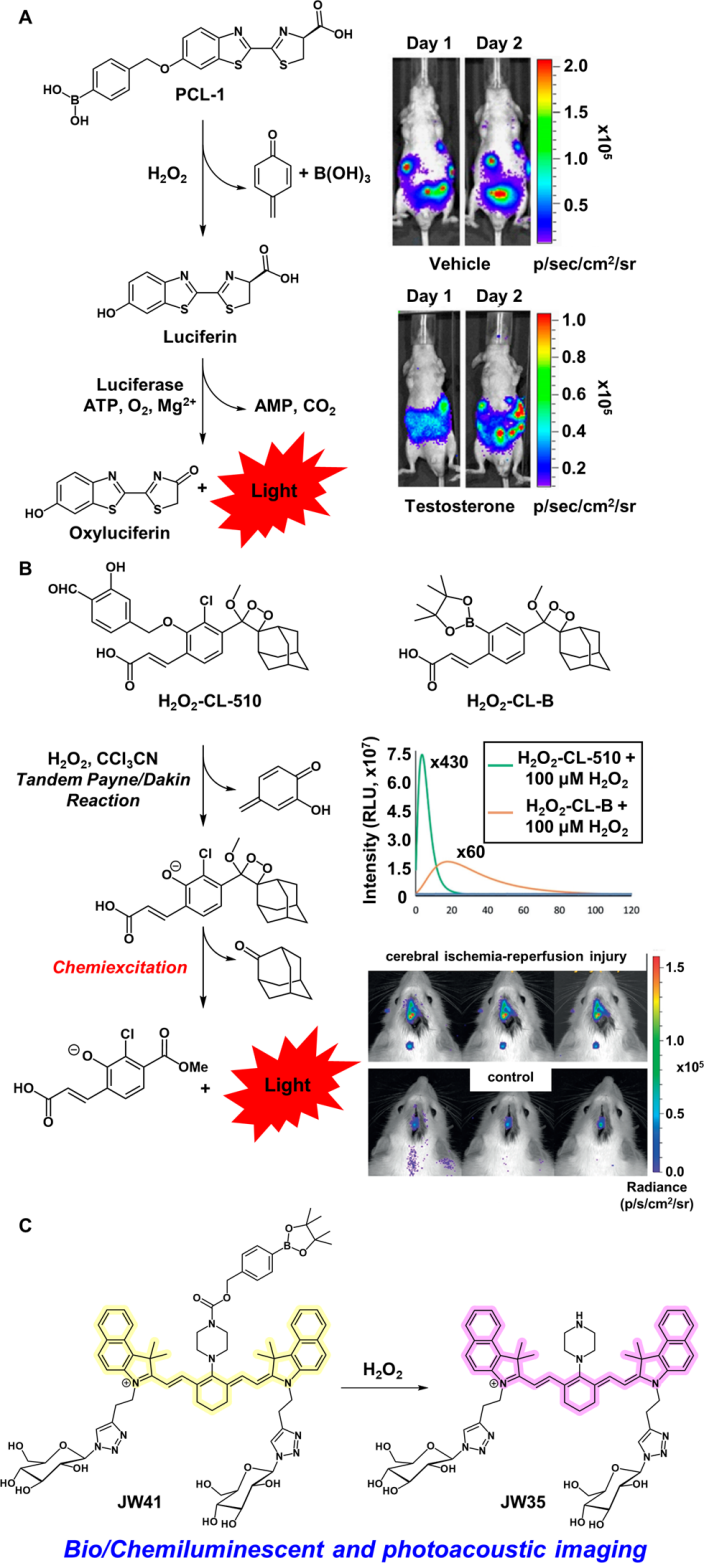
(A) H_2_O_2_-mediated uncaging of PCL-1 and its use in testosterone stimulated LNCap-luc tumor xenograft mouse models. Adapted with permission from ref (113). Copyright 2010 National Academy of Sciences. (B) H_2_O_2_-mediated uncaging process for chemiluminescence ABS probes, luminescence intensity changes upon uncaging, and their use in mouse models of cerebral ischemia -reperfusion injury. Adapted with permission from ref (111). Copyright 2020 Wiley. (C) Scheme depicting H_2_O_2_-mediated uncaging of JW41 to JW35 which allows for photoacoustic and fluorescence dual-modal imaging in vivo and in excised fixed tissue.

As an introduction to bioluminescent-based H_2_O_2_ reporting strategies for use in animal models, our laboratory synthesized Peroxy Caged Luciferin-1 (PCL-1, Figure 6A).^113^ PCL-1 is a firefly luciferin analogue caged with a self-immolative benzyl boronic acid group. PCL-1 reports on endogenous H_2_O_2_ variations via a bioluminescent readout generated from the reaction of firefly luciferin with the luciferase enzyme in the presence of ATP and Mg^2+^. PCL-1 was able to monitor H_2_O_2_ production in both live luciferase expressing mice (FVB-luc+) and in a testosterone stimulated LNCap-luc tumor xenograft model in immunodeficient SCID hairless outbred (SHO) mice.^113^ We also developed a dual-analyte responsive system composed of Peroxy Caged Luciferin-2 (PCL-2) which releases hydroxy-cyanobenzothiazole upon H_2_O_2_ sensing and the pentapeptide z-Ile-Glu-Thr-Asp-d-Cys (IETDC) that releases free d-cysteine upon reaction with caspase-8 (Figure 2).^96^ Caspase-8 plays a prominent role in initiating apoptotic pathways during inflammatory signaling cascades. In situ formation of firefly luciferin via condensation of d-cysteine and hydroxy-cyanobenzothiazole in aqueous solutions provides a bioluminescent reporter when coupled with luciferase systems. Thus, this strategy reports on elevations in both caspase-8 and H_2_O_2_ but is effectively shut off in the presence of only one analyte or none, akin to a molecular AND-type logic gate. The PCL-2 and IETDC reporting strategy was enabled the *in vivo* detection of endogenous elevations of both H_2_O_2_ and caspase-8 in LPS treated mice modeling acute inflammatory responses^96^ and has been expanded to other activity-based bioluminescent reporters.^115^

It is also worth noting chemiluminescent reporters which preclude the need for luciferase expressing systems.^116^ Many such probes pioneered by the Shabat laboratory in particular, utilize Schaap’s dioxetane fragments to generate bright luminescent readouts.^111,112,117−120^ In a recent report, they synthesized both H_2_O_2_-CL-510 and H_2_O_2_-CL-B as chemiluminescent reporters on H_2_O_2_ fluxes (Figure 6B). H_2_O_2_-CL-B makes use of the aryl boronate ester trigger but exhibits a much weaker turn-on response than H_2_O_2_-CL-510 which contains a salicylaldehyde trigger that undergoes an H_2_O_2_-mediated Payne/Dakin reaction cascade to provide a highly emissive (×430) turn-on response (Figure 6B). As such, H_2_O_2_-CL-510 was carried forward in studies monitoring H_2_O_2_ bursts due to cerebral ischemia-reperfusion injury in rat models (Figure 6B).^111^ While salicylaldehyde-based probes offer a promising alternative to aryl boronate triggers for H_2_O_2_ sensing, the need for a trichloroacetonitrile additive to promote uncaging may preclude its use in some biological models.

Photoacoustic (PA) imaging has garnered significant interest for *in vivo* imaging applications as it utilizes the detection of sound via probe excitation with longer wavelength deeper penetrating light.^121^ Acoustic detection enables depth penetration at centimeter length scales as sound scatters significantly less than emitted light in biological tissues.^121,122^ Photoacoustic probes are generally designed to minimize nonradiative decay processes thereby ensuring the majority of energy loss upon irradiation results in the generation of heat as opposed to fluorescence emission. The increase in temperature causes thermoelastic expansion in the surrounding tissues and the pressure changes result in the generation of sound waves which provide the image contrast.^121^ Acoustogenic probes using the photoacoustic modality provide a promising direction for the activity-based sensing field.^123^

In the context of activity-based H_2_O_2_ sensing, Bohndiek and co-workers developed JW41 which leverages a heptamethine carbocyanine core scaffold with 2-deoxyglucose targeting groups and a centrally linked aryl boronate cage, for dual modal photoacoustic and fluorescence imaging (Figure 6C).^110^ The pendant glucose groups enhance uptake in cancerous tissues which generally exhibit higher levels of oxidative stress. Caged JW41 displays an absorption maximum at 730 nm that shifts to 790 nm upon uncaging to JW35. Though the fluorescence maximum centered at 825 nm is identical in both forms, there is a significant 100% increase in the fluorescence quantum yield of the uncaged dye. This dye was amenable to *in vitro* fluorescence imaging of H_2_O_2_ in MDA-MB-231 and MCF7 cells. Importantly, intravenous injection of JW41 enabled PA imaging of H_2_O_2_ elevations in subcutaneous MDA-MB-231 tumors in nude mice. While JW41 localized to both the tumors and liver, oxidative uncaging was substantially higher in tumors. *Ex vivo* fluorescence imaging of fixed tumor sections was also carried out and demonstrated cytosolic probe localization with substantially decreased accumulation in necrotic tumor regions.^110^ Additionally, the Hai group has also developed an elegant strategy for mitochondrial-targeted dual photoacoustic and fluorescence imaging applied to *in vivo* mouse models of inflammation.^109^

## Tandem Activity-Based Sensing and Labeling Probes to Enable Cell-Specific Profiling

The development of tandem activity-based sensing and labeling strategies to create cell-trappable molecular probes has recently garnered significant research interest. Probes described in this section undergo H_2_O_2_-mediated uncaging to release either a molecule or reactive intermediate capable of labeling proximal internal cellular structures. Such probes greatly enhance signal-to-noise responses, preserve spatial information, and offer key insights into transcellular signaling processes. The new biological information revealed by such chemical reagents contrasts with many of the fluorophores discussed in previous sections, which have significantly advanced our understanding of intracellular H_2_O_2_ signaling. We also differentiate tandem ABS and labeling probes from previously described organelle-targeting fluorophores in that the localization of the probe is dictated by reactivity as opposed to an orthogonal specialized targeting group and/or esterase-mediated uncaging.

**Figure 7:**
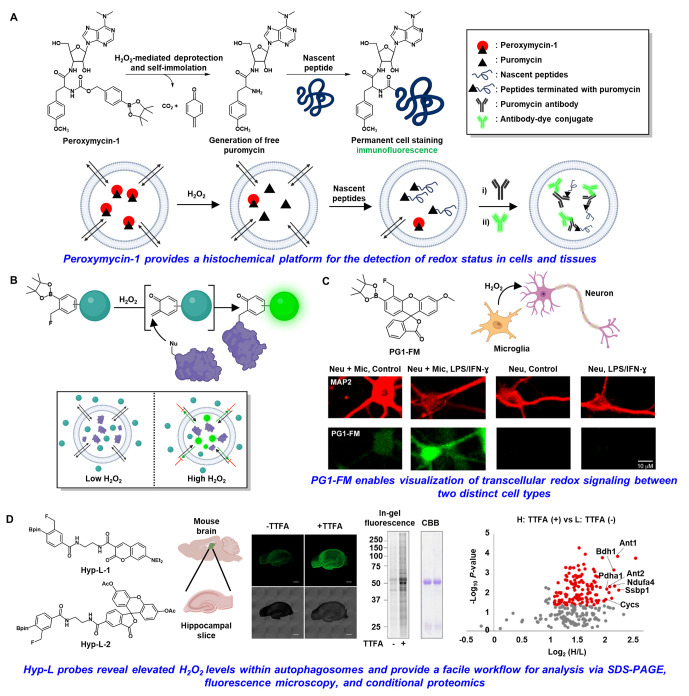
(A) Schematic and cartoon depicting the workflow for the peroxymycin-1 histochemical approach toward H_2_O_2_ detection. (B) Schematic demonstrating the H_2_O_2_-responsive quinone methide fluorophore trapping strategy. (C) PG1-FM enabled the visualization of transcellular H_2_O_2_ signaling in a microglia-neuron coculture model. Fluorescence was observed only in microglia-neuron cocultures stimulated with LPS/IFN-γ but not in neuron monocultures treated with LPS/IFN-γ. Adapted with permission from ref (133). Copyright 2021 National Academy of Sciences. (D) Structures of Hyp-L-1 and Hyp-L-2 and their use in proximity labeling under oxidative conditions for analysis via fluorescence confocal imaging, SDS-PAGE, and conditional proteomics. Adapted from ref (38). Copyright 2020 American Chemical Society.

The first tandem ABS and labeling strategy for H_2_O_2_ developed in our laboratory relied on immunodetection of the aminonucleoside puromycin as a histochemical readout.^124,125^ Puromycin is an aminoacylated-tRNA analogue that inhibits protein translation at the ribosome and is terminally incorporated into nascent peptides and proteins. Taking inspiration from a previously reported collaboration with the Renslo group, we synthesized Peroxymycin-1, a puromycin caged with an aryl boronate ester trigger at the α-amino position necessary for peptide bond formation (Figure 7A).^124,126^ H_2_O_2_-mediated uncaging results in a self-immolation cascade generating free puromycin that, when incorporated into nascent biomolecules, results in a permanent and dose-dependent label that can be visualized through immunofluorescence using puromycin-specific antibodies (Figure 7A). Notably, the amenability of this strategy toward biological fixation complements the many existing ABS fluorophores discussed in this review, which may degrade under conditions necessary for sample fixation.

The covalent nature of the cell-trapping mechanism inherent to the puromycin-based approach allows for significantly increased sensitivity over previously reported ABS and organelle-targeted ABS fluorophore derivatives. The considerable sensitivity facilitated the profiling of basal H_2_O_2_ levels across a panel of cancer and nontumorigenic breast cell lines and revealed heightened H_2_O_2_ levels in metastatic cancer cells compared to less invasive and nontumorigenic cells. This work reveals a correlation between basal H_2_O_2_ levels and cellular metastatic potential. Deployment of this puromycin-based strategy in mouse tissue models of diet-induced nonalcoholic fatty liver disease displayed increased levels of oxidative stress in liver tissue lysates from mice fed a high-fat diet vs a normal diet. These results agreed with similar experiments employing 4-hydroxy-2-nonenal (4-HNE) immunostaining indicating the complementarity of puromycin-based approaches to more widely used methods, however we note that 4-HNE is not a selective reporter of H_2_O_2_ but an indirect proxy for oxidative stress.^124,125,127^

As the α-amino group on puromycin is a general synthetic handle that can undergo modification with a range of specialized ABS triggers, we envision a wide range of opportunities to employ puromycin and other histochemical scaffolds to monitor other dynamic biological analytes. In this context, our laboratory is actively engaged in expanding ABS-mediated intracellular fluorophore trapping strategies to include proximity labeling via the generation reactive intermediates upon analyte detection. Such methods complement the puromycin-based platforms that harness cellular machinery to become intracellularly trapped. Indeed, metal-directed acyl-imidazole (MDAI) chemistry as an intracellular trapping strategy has advanced our understanding of cellular signaling processes mediated by redox active and labile transition metals.^128−130^ In this design, an acyl imidazole fragment bridges both a metal-sensing motif and a fluorophore. Reversible metal chelation enhances the electrophilicity of the acyl imidazole unit thereby driving biomolecular labeling forward.^128−130^

In another example of tandem activity-based sensing and labeling, we were inspired by recent reports from Urano and co-workers, where they utilized *o*-quinone methide chemistry to promote cell retention of β-galactosidase substrates.^131,132^ We reasoned that *o*-quinone methide chemistry provides an excellent blueprint for the H_2_O_2_-driven proximity labeling and cell trapping of fluorophores. To this end, we developed PeroxyGreen-1 Fluoro Methyl (PG1-FM) as a first-generation analogue for tandem ABS and labeling (Figure 7B, C).^133^ Key to this strategy is the installment of a fluoromethylene unit proximal to the aryl boronate ester trigger on the fluorescein core scaffold. H_2_O_2_-mediated uncaging and subsequent fluoride elimination results in the generation of a highly reactive *o*-quinone methide intermediate that undergoes nucleophilic biomolecule labeling to produce a trapped fluorescent readout (Figure 7B, C).^133^

PG1-FM enabled visualization of endogenous H_2_O_2_ elevations in live-cell models of oxidative stress and redox signaling. Notably, unlike parent analogues, a strong fluorescence signal is retained in PG1-FM stained cells after multiple washing steps.^55,133^ Additionally, in-gel fluorescence analysis from sodium dodecyl sulfate–polyacrylamide gel electrophoresis (SDS-PAGE) of PG1-FM stained cell lysate treated with H_2_O_2_ revealed proteome-wide labeling.

To move beyond exploring the intracellular signaling landscape, we sought to harness tandem ABS and labeling approaches to map cell-to-cell redox signaling processes. In collaboration with the Swanson group, we deployed PG1-FM to study transcellular redox signaling in a microglia-neuron coculture system. This system models neuronal injury mediated by microglial activation, which is the hallmark of neural degenerative disorders such as Alzheimer’s and Parkinson’s. Microglia can be activated to produce H_2_O_2_ via treatment with lipopolysaccharide (LPS) and interferon-gamma (IFN-γ); however, LPS and IFN-γ do not have any effect on ROS production in neurons (Figure 7C). We showed that neurons cocultured with microglia and treated with LPS and IFN-γ exhibited elevations in H_2_O_2_ whereas neuron monocultures treated under the same conditions do not. Additional coculture studies employing p47^pho^ knockout (p47^phox–/–^) microglia cells exhibit attenuated H_2_O_2_ production when treated with LPS and IFN-γ, thus indicating that PG1-FM is selectively capturing H_2_O_2_ fluxes that are produced in microglia and travel to neighboring neurons. Taken together, these experiments establish that PG1-FM is capable of identifying transcellular H_2_O_2_ redox signaling with single-cell resolution in complex coculture models, opening the door to studies of ROS-mediated cell–cell communication (Figure 7C).^133^

In parallel to our investigations, the Hamachi group reported a similar H_2_O_2_-responsive *o*-quinone methide labeling strategy.^38^ This impressive work introduced both coumarin (Hyp-L-1) and fluorescein (Hyp-L-2) derivatives in which the ABS and labeling fragment was tethered to the dye as opposed to attached directly onto the main fluorophore scaffold (Figure 7D). Intracellular biomolecule labeling under oxidative conditions using the Hyp-L workflow enabled analysis via SDS-PAGE, fluorescence confocal microscopy, and conditional proteomics. Hyp-L-2 was used for their key studies which involved fluorescence confocal imaging of fixed hippocampal slices from mouse brain tissues under 2-thenoyltrifluoroacetone (TTFA) stimulated conditions (Figure 7D).^38^ TMT-based quantitative proteomic analysis of mouse brain tissues either treated with TTFA or not and incubated with Hyp-L-2 revealed H_2_O_2_-induced proximity labeling of predominately mitochondrial proteins which correlates with the pharmacological mechanism of TTFA which promotes oxidative stress by inhibiting mitochondrial complex II. Additionally, this work provides a significant advance in our understanding of H_2_O_2_-induced autophagy as the authors identified autophagosomes enriched with H_2_O_2_ in PMA stimulated RAW264.7 macrophages utilizing the Hyp-L-2 microscopy and proteomic workflow.^38^

## Outlook and Future Directions

Activity-based sensing (ABS) provides a general and versatile platform for chemistry-enabled advances in biology. In particular, small-molecule probes offer a number of advantages for bioimaging, including their ease of use, highly tunable nature, and amenability to rapid screening across multiple biological models. Indeed, the swift development of synthetic small-molecule ABS fluorophores parallels that of genetically encodable protein redox sensors, which often demonstrate unrivaled site specificity and sensitivity. The prevailing spirit of the field is not one of competition but instead the bolstering of available resources and tools that enhance our abilities to draw the most valid conclusions from the model being studied, especially when such tools are deployed in tandem. Thus, there exists plenty of opportunities for expansion and improvements on both fronts.

There is an ongoing need to discover new triggers that exhibit more rapid uncaging kinetics relative to the archetypical aryl boronate ester scaffold.^1^ Exciting work by Vauzeilles and colleagues on borinic-acid-based coumarin dyes shows an impressive second-order rate constant of 1.9 × 10^4^ M^–1^·s^–1^ toward H_2_O_2_-mediated oxidation.^134^ The replacement of one oxygen linkage with carbon decreases electron donation into the empty boron p-orbital, thereby increasing electrophilicity at that position.^135^ This substitution results in an observed 10 000-fold rate enhancement over aryl boronic ester triggers and showcases the effect that seemingly subtle synthetic manipulations have on ABS fluorophore systems. The reported borinic-acid-based coumarin was selective for H_2_O_2_ over other ROS and exhibited a significantly faster turn-on response in live COS7^gp91/p22^.^134^ Recent follow-up work detailed the synthesis of a more general trigger derivative that was further modified to overcome challenges related to the regioselectivity of oxidative deprotection.^136^ Another direction is in catalytic activity-based sensing where one uncaging event can lead to multiple signal outputs. This strategy is highlighted by elegant work from Shabat and colleagues on self-immolative polymer scaffolds that can be uncaged by a single boronate oxidation or systems by Phillips that self-sustain peroxide consumption and generation for continuous detection.^137−139^ Indeed, the development of ABS triggers with faster uncaging kinetics holds potential for offering enhanced spatial resolution. When coupled with new tandem sensing and labeling approaches, such ABS tools provide a platform for activity-based proximity labeling and profiling of key redox regulators akin to the activity-based protein profiling (ABPP) approaches pioneered by the Cravatt and Bogyo laboratories and many other prominent groups.^140−142^ The trade-off for heightened reactivity is often diminished ROS selectivity, and thus great care should be taken when designing experiments and when attributing the tandem ABS and labeling process to a specific ROS.^9,143^

In parallel, improvements in small-molecule H_2_O_2_ sensors can also rely on the application of constantly evolving design principles of the parent fluorophore structures responsible for photophysical and chemical characteristics.^144−146^ The introduction of new dyes with specially tailored equilibrium constants between emissive “open” and nonemissive “closed” forms that are amenable to emergent single-molecule localization microscopy (SMLM) techniques provides extensive starting points to explore ABS systems with drastically enhanced resolution over diffraction limited microscopy techniques.^146,147^ Additionally, the development of far-red (700–780 nm) and short-wave infrared (SWIR, 1000–2000 nm) shifted dyes is of extreme importance as the longer wavelength absorption/emission profiles enhance depth penetration and minimize background autofluorescence from biological structures thus greatly enhancing *in vivo* bioimaging.^148−153^ As such, ample opportunities remain to leverage the rapidly growing toolbox of ABS triggers with synthetic advances in fluorophore structure design. The continued evolution of which coupled with translation into increasingly complex biological models will open new and exciting avenues to further our understanding of basic biology and translation to diagnostics and medicines. Indeed, the area of activity-based diagnostics is an exciting direction in this burgeoning field.^154^
